# A critical assessment of gene catalogs for metagenomic analysis

**DOI:** 10.1093/bioinformatics/btab216

**Published:** 2021-04-01

**Authors:** Seth Commichaux, Nidhi Shah, Jay Ghurye, Alexander Stoppel, Jessica A Goodheart, Guillermo G Luque, Michael P Cummings, Mihai Pop

**Affiliations:** Center for Bioinformatics and Computational Biology, University of Maryland, College Park, MD, 20742, USA; Biological Science Graduate Program, University of Maryland, College Park, MD, 20742, USA; Division of Molecular Biology, Office of Applied Research and Safety Assessment, Center for Food Safety and Applied Nutrition, U.S. Food and Drug Administration, Laurel, Maryland, 20708, USA; Center for Bioinformatics and Computational Biology, University of Maryland, College Park, MD, 20742, USA; Department of Computer Science, University of Maryland, College Park, MD, 20742, USA; Center for Bioinformatics and Computational Biology, University of Maryland, College Park, MD, 20742, USA; Department of Computer Science, University of Maryland, College Park, MD, 20742, USA; Center for Bioinformatics and Computational Biology, University of Maryland, College Park, MD, 20742, USA; Scripps Institution of Oceanography, University of California, San Diego, La Jolla, CA, 92037, USA; Department of Microbiome Science, Max Planck Institute for Developmental Biology, Tübingen, 72076, Germany; Center for Bioinformatics and Computational Biology, University of Maryland, College Park, MD, 20742, USA; Center for Bioinformatics and Computational Biology, University of Maryland, College Park, MD, 20742, USA; Department of Computer Science, University of Maryland, College Park, MD, 20742, USA

## Abstract

**Motivation:**

Microbial gene catalogs are data structures that organize genes found in microbial communities, providing a reference for standardized analysis of the microbes across samples and studies. Although gene catalogs are commonly used, they have not been critically evaluated for their effectiveness as a basis for metagenomic analyses.

**Results:**

As a case study, we investigate one such catalog, the Integrated Gene Catalog (IGC), however, our observations apply broadly to most gene catalogs constructed to date. We focus on both the approach used to construct this catalog and on its effectiveness when used as a reference for microbiome studies. Our results highlight important limitations of the approach used to construct the IGC and call into question the broad usefulness of gene catalogs more generally. We also recommend best practices for the construction and use of gene catalogs in microbiome studies and highlight opportunities for future research.

**Availability and implementation:**

All supporting scripts for our analyses can be found on GitHub: https://github.com/SethCommichaux/IGC.git. The supporting data can be downloaded from: https://obj.umiacs.umd.edu/igc-analysis/IGC_analysis_data.tar.gz.

**Supplementary information:**

Supplementary data are available at *Bioinformatics* online.

## 1 Introduction

Increasingly, studies of microbial communities rely on metagenomics—the sequencing of DNA extracted directly from a microbial mixture. Assembling metagenomic reads into longer contiguous sequences (contigs) is still a computationally challenging problem, because of repeated sequences within and among genomes, uneven abundances of organisms, sequencing errors and strain-level variation. Due to these challenges, and to limitations of sequencing technology, reconstructing complete and accurate genomes for all organisms in a single, complex metagenomic sample is still challenging. Given enough samples, metagenome assembled genomes can be reconstructed for many, but often not all, of the species comprising a microbiome. Regardless, metagenomic assemblies typically comprise many small contigs of unknown taxonomic origin.

The fragmented nature of metagenomic assemblies complicates data analysis, both because it is difficult to associate genomic fragments with individual taxa, and because it is difficult to identify related genomic fragments across samples. For these reasons, the earliest metagenomic studies focused on genes (and their inferred functions) found within assembled fragments, ignoring their precise taxonomic origin. Even in fragmented data, genes can be fairly effectively identified (Rho *et al*., 2010). A gene-centric approach was used in the first large scale metagenomic study of ocean bacteria (Yooseph *et al*., 2007). To prevent overcounting due to sequencing and assembly errors, or due to small differences in gene sequences within closely related organisms, Yooseph *et al.* (2007) clustered the protein sequences based on similarity and focused their analysis on the representative sequence of each cluster. This gene ‘catalog’ revealed the tremendous diversity of bacterial functions in the ocean, with the newly predicted protein sequences doubling the number of known proteins. The MetaHIT project (Qin *et al.*, 2010) constructed a similar catalog in order to characterize the functional composition of the human gut microbiome. Qin *et al*. (2012) leveraged a gene catalog as the basis for a microbiome association study in type 2 diabetes, and introduced the concept of metagenomic linkage groups—groups of genes that co-vary in abundance across samples. The gene catalog thus represents the basis for grouping together genes that likely originate from a single organism, an idea further extended by Nielsen *et al.* (2014) to help reconstruct partial genome sequences from metagenomic data.

Following these initial studies, gene catalogs have become ubiquitous in the analysis of metagenomic datasets, and have been created for the gut microbiota of multiple animals [e.g. mouse (Xiao *et al*., 2015), rat (Pan *et al*., 2018), pig (Xiao *et al*., 2016a, b), dog (Coelho *et al.*, 2018), cow (Li *et al*., 2020), macaque (Li *et al*., 2018), chicken (Huang *et al*., 2018), lion, leopard and tiger (Mittal *et al*., 2020)], ocean bacteria (Sunagawa *et al.*, 2015), soil bacteria (Lou *et al*., 2019) and the human vagina (Ma *et al*., 2020) and respiratory tract (Dai *et al*., 2019). Gene catalogs are commonly used to: (i) reduce redundancy in the data, thereby improving estimates of diversity (Yooseph *et al.*, 2007); (ii) act as a common frame of reference across samples and studies; (iii) serve as a basis for metagenomic-wide association studies (Wang and Jia, 2016); and (iv) guide the binning of metagenomic contigs into organism-specific groups ( Nielsen *et al*., 2014; Plaza Oñate et al., 2018).

Such analyses may be confounded by the specific properties of the catalog being used. Yet, to our knowledge, the structure and construction of gene catalogs have not been critically evaluated. Because the processes for constructing and using gene catalogs are broadly the same across studies, generalizable observations can be obtained from the analysis of any of the catalogs referenced above. We focus here on the Integrated Gene Catalog (IGC) (Li *et al.*, 2014), which seeks to provide a nearly comprehensive collection of the gene sequences identified in the human gut microbiome. We chose the IGC because it provides all the supporting metadata and intermediate files necessary to conduct a critical analysis of the structure of the resulting clusters.

### 1.1 The construction and use of gene catalogs

Catalog construction starts by identifying genes within metagenomic data. The gene sequences are then clustered together based on similarity in order to remove trivial differences between sequences due to fragmentary data (e.g. genes that miss the start or stop codons), sequencing errors or small, strain-level variations. The clustering can be performed at the DNA level [e.g. the IGC (Li *et al.*, 2014)], or at the amino acid level [e.g. the Global Ocean Survey (Yooseph *et al.*, 2007)]. Analysis at the DNA level provides greater resolution for taxonomic classification, whereas the amino acid level is better suited for functional analysis and is more able to group together distantly related but functionally similar sequences. The implied, but often unstated, goal of the clustering process is to reproducibly group together sequences that have the same function and/or taxonomic origin, thereby defining the gene from which the sequences are derived in a way that is consistent across samples. Each cluster is typically represented by one sequence, either a representative selected from the sequences clustered together, or a sequence that represents the consensus of the clustered sequences. Beyond the obvious use of these sequences in a broad range of sequence-based analyses (e.g. database searches, function or structure prediction), the cluster representatives can also be used to estimate the relative abundance of the corresponding genes within microbiome samples.

### 1.2 Historical context

Clustering of biological sequences that share a common function or taxonomic origin has been at the core of biological research long before the first metagenomic experiment. Databases such as the Clusters of Orthologous Groups (COG) (Tatusov *et al*., 2000) and Pfam (Sonnhammer *et al*., 1997) date back to the late 1990s and were developed to organize the rapidly accumulating protein sequence information. To define the boundary of clusters, these databases used reciprocal best hit links (COG), or hidden Markov models built upon multiple alignments of related proteins (Pfam), approaches that rely on statistical significance measures instead of arbitrary thresholds based on sequence similarity. At the same time, taxonomic analyses based on housekeeping genes relied on careful phylogenetic analyses to define species boundaries (Lan and Reeves, 2001).

In the early 2000s, metagenomic studies yielded much larger datasets than previously seen. The challenge of effectively scaling analyses to cope with increasingly larger datasets led to the development of new approaches that emphasized speed over the accuracy or comprehensiveness of the analysis. CD-HIT (Li and Godzik, 2006), for example, a greedy clustering approach we briefly describe below, was developed to address the challenges encountered when analyzing the data from the Global Ocean Survey. Although CD-HIT and some other clustering tools developed (Edgar, 2010; Ghodsi *et al*., 2011) relied on fixed thresholds to determine the boundaries of clusters, it was already recognized that such thresholds were not consistent with biologically relevant entities (Nguyen *et al*., 2016; Shah *et al*., 2018); for a given threshold, some clusters contained sequences from multiple species, whereas other species were represented in multiple clusters.

The estimation of abundances from sequencing reads is a relatively new development in metagenomic studies but has been used extensively in the study of gene expression in eukaryotes. A number of factors have been identified that confound abundance estimation including multi-mapped reads, uneven depth of coverage, and sequence composition biases. Computational and statistical approaches have been developed to address such challenges (Bray *et al.*, 2016; Li and Dewey, 2011; Patro *et al*., 2014, 2017).

### 1.3 Overview of the integrated gene catalog

The Integrated Gene Catalog comprises 9 879 896 annotated gene clusters that were constructed from a combination of 511 prokaryotic reference genomes from species known to occur in the human gut, and 1267 gut metagenome datasets from Chinese, American and European cohorts. The IGC has been used to discover correlations between gut microbiome composition and resistance to immune checkpoint inhibitors in cancer patients (Routy *et al*., 2018), to observe that microbiome composition is modulated to a greater degree by environmental factors than by human genetics (Rothschild *et al*., 2018), to correlate glycemic response after meals with microbiome composition (Zeevi *et al*., 2015) and to identify signs of human fecal contamination in a river with sewage input (Meziti *et al*., 2016).

The IGC was created through a multistep clustering process (Li *et al*., 2020). First, separate gene catalogs were created from the metagenomic data derived from each cohort: American (AGC), Chinese (CGC) and European (EGC), and for the sequenced prokaryotic reference genomes collection (SPGC). The three cohort-specific gene catalogs were then clustered together into a larger gene catalog called the 3CGC, which was then clustered with the SPGC catalog to create the IGC. Gene clustering was performed with CD-HIT (Li and Godzik, 2006). As employed in the construction of the IGC, this tool operates in an iterative fashion, processing the gene sequences in decreasing order of length. The longest gene sequence is selected to be the representative of the first cluster. The next longest sequence is then assigned to the cluster if it matches the representative sequence with ≥95% sequence identity over ≥90% of the length of the query sequence, or becomes the representative of a new cluster. In the following iterations, query sequences either become representatives of new clusters or are added to an existing cluster if they match the corresponding representative sequence sufficiently well. For most applications, only the set of representative sequences is used, however, the IGC project also provides the full assignment of individual genes to clusters. Each representative gene sequence in the IGC is assigned, if possible, taxonomic and functional labels, however, only 16.3% of the sequences are assigned a genus-level annotation and only 60.4% have functional annotations.

## 2 Results

### 2.1 Inconsistent fidelity of clustering

**Fig. 1.**
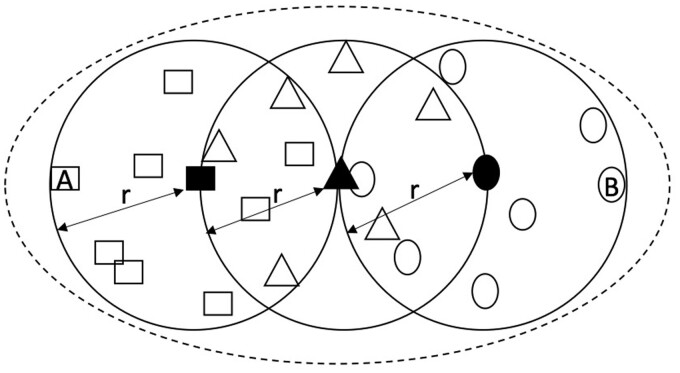
Transitive clustering error. The circles represent three clusters from three distinct catalogs. Within each catalog, the sequences within a cluster (represented by points of different shapes) are guaranteed to be within a distance r (5% divergence in the case of the IGC) from the corresponding cluster representative (solid shape). When merging multiple catalogs, only the representative sequences are clustered together (also within the same tolerance r), while the sequences contained within each cluster are implicitly assigned to the same cluster as the corresponding representative. In this figure, after clustering the representatives in one round, the triangle cluster representative is the representative of a meta-cluster (dashed line) that includes the representative sequences of the square and circle clusters. Within this cluster, the maximum distance between two sequences (marked with A and B in the figure), may be as high as 4r, or 20% sequence divergence in the case of the parameters used in the IGC. The distance between a sequence and its corresponding cluster representative may be as high as 2r, or 10% sequence divergence

That a 95% sequence identity cut-off is used throughout the multiple rounds of clustering in the construction of the IGC appears to imply that the final clusters are consistent with this threshold. However, the multiple rounds of clustering used to construct the IGC may yield clusters with a (much) lower identity than the intended threshold. We call this methodological artifact transitive clustering error (Fig. 1), which occurs when different gene catalogs are sequentially clustered. Although each clustering step guarantees the 95% threshold for the sequences being clustered, this threshold does not constrain the similarity between sequences that were clustered in prior iterations. The result of transitive clustering error is an unintended increase in the effective radius of the new cluster with respect to the representative sequence (see Supplementary File S1 for a detailed explanation of transitive clustering error). When the three cohorts were clustered into the 3CGC, individual gene sequences could potentially share as low as 90% identity to the new representative gene sequences, while two sequences within a cluster may share as little as 80% sequence identity. The final clustering of the SPGC and the 3CGC, could potentially have clustered sequences with only 85% identity to the representative sequence and as low as 70% identity between sequences assigned to the same cluster.

**Fig. 2.**
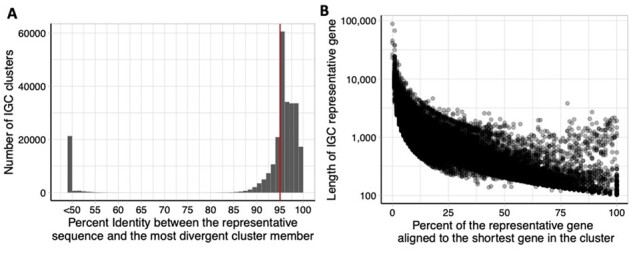
Undesirable effects of gene clustering in the IGC. Data shown refer to the 255 191 IGC clusters that contain 100 sequences or more. (**A**) The distribution of CD-HIT percent identity between the representative and the most divergent cluster member. The vertical red line indicates the 95% identity clustering threshold used to create the IGC. Note that many sequences are below the target threshold of 95%. (**B**) Relationship of percent of the representative gene aligned to the shortest cluster member and the length of the representative gene

To evaluate the actual impact of transitive clustering error within the IGC, we focused on the 255 191 IGC gene clusters that contained at least 100 sequences each. Among these clusters, 29.6% contained sequences that differed from the cluster representative by more than the intended 95% identity cut-off (Fig. 2A). Furthermore, 8.2% of the clusters contained sequences that are different by 50% or more from the corresponding cluster representative. This difference is much higher than the expected error due to transitive clustering. An explanation is that the construction of the IGC did not require full length alignments to each cluster representative, but rather allowed matches that cover as little as 90% of the clustered sequence. In the worst case, after two or more rounds of clustering, sequences within an IGC cluster may not overlap with the selected representative sequence at all (Supplementary Fig. S1).

The process used to construct the IGC does not constrain the fraction of the representative sequence that needs to match the sequences within the cluster. This choice makes it possible for two sequences to both align to the cluster representative perfectly without sharing any sequence with each other. As an example, cluster 303 contains four sequences of different lengths—16 111 nt (representative), 7122 nt, 3012 nt and 2982 nt. All of these genes are complete, spanning from start codon to stop codon and originate from the SPGC (genes found in nearly complete reference genomes). The alignment between the three genes to the cluster representative (Supplementary Fig. S2) demonstrates the lack of overlap between the individual sequences, suggesting that they align to distinct domains of the representative sequence, rather than representing variants of this gene. Supplementary Table S1 lists the domains found in the representative sequence. This artifact may be wide-spread within the IGC—within the 255 191 IGC clusters with a minimum of 100 members, the mean difference between longest and shortest gene length is 590 nt, representing an average of 14.4% of the length of the cluster representative (Fig. 2B).

### 2.2 Taxonomic inconsistency of clusters

The 95% identity threshold selected by the IGC was intended to create clusters with taxonomic homogeneity at the species level (Li *et al*., 2014). Taxonomic homogeneity is desirable for analyses with the IGC, however, as we briefly described above, it has long been recognized that no specific threshold can universally and accurately capture biologically meaningful boundaries (Nguyen *et al.*, 2016; Shah *et al.*, 2018).

This can be demonstrated by clustering the genes of genera like *Bacteroides* and *Lactobacillus* comprising multiple species within which many strains have been sequenced. We separately clustered the RefSeq genes from 167 *Bacteroides* (5 355 696 genes) and 166 *Lactobacillus* species (1 876 284 genes) using the IGC clustering parameters. Of the resulting 438 106 *Bacteroides* and 256 949 *Lactobacillus* clusters, 32% and 24% were composed of multiple species, respectively.

The taxonomic homogeneity of the IGC clusters can be most readily assessed within the SPGC because this gene catalog has well-defined taxonomic labels; however, we note that the SPGC only contains 200 species with sparse representation per species (a mean of 2.6 reference genomes). Still, we found that 42 208 (6.4%) of all clusters in the SPGC grouped together sequences from multiple distinct species, with a maximum of 21 species in a single cluster.

**Fig. 3.**
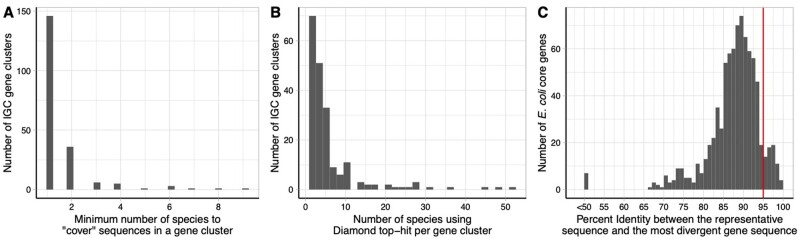
IGC clusters can contain more than one species. A and B show the taxonomic heterogeneity of 200 IGC clusters (the 100 largest clusters and 100 randomly chosen clusters with at least 100 sequences). (**A**) The minimum number of species such that each sequence in a cluster had at least one significant Diamond hit to one of these species (**B**) Number of species per cluster if each sequence is assigned the label of the top Diamond result. (**C**) The distribution of CD-HIT percent identity between the representative and the most divergent gene sequence for the 818 core genes of *E.coli* identified from 86 830 assemblies. The red vertical line denotes 95% identity, the IGC clustering threshold

To estimate the number of species within the IGC clusters derived from sequences with unknown taxonomic origin (namely, the three country-specific catalogs), we focused on a subset of 200 IGC clusters: the 100 largest clusters and 100 randomly chosen clusters from those with at least 100 sequences each. We aligned each sequence within an IGC cluster to the NCBI nr database (version 5) using Diamond (Buchfink *et al*., 2015) (version 0.9.29). We used the same alignment thresholds as those used by the IGC, requiring at least 95% sequence identity and 90% query coverage. We retained all database entries that matched each query sequence within these thresholds. We conservatively inferred the number of species per gene cluster using a minimum set cover approach. Specifically, we identified the smallest number of species such that each sequence had at least one hit to a database sequence from one of these species. As seen in Figure 3A, 73% of clusters (57% of the largest and 89% of the randomly selected clusters) are covered by a single species. If we used just the top database hit for each sequence, the most commonly used approach in practice, only 20.5% of clusters (5% of the largest and 36% of the randomly selected clusters) were composed of a single species (Fig. 3B).

To explore the converse, the possibility that variants of a gene from a single species may be distributed across multiple clusters, we analyzed a collection of 86 830 *Escherichia coli* genomes obtained from the GenomeTrakr database (Allard *et al*., 2016). When focusing on just the 818 core genes of the *E.coli* pan-genome (genes found in all of the genomes), the mean sequence identity between the representative and the most divergent clustered sequence was 87.7% which is lower than the 95% threshold used by the IGC. In fact, only 63 core genes met or exceeded the 95% threshold and would have been clustered properly by the IGC (Fig. 3C).

### 2.3 Hidden species within the IGC

A direct consequence of multi-species clusters is the possibility that genes from an individual species may be ‘hidden’ by representative sequences belonging to a different species. A species for which no gene is selected as a representative for a cluster in the catalog becomes effectively undetectable in the samples being analyzed.

To explore the extent of this problem, we focused on just the SPGC (genes from complete and near complete genomes) because these genes have well defined taxonomic labels. Within the SPGC, the number of representative genes per species ranged from 139 (*Escherichia* sp. 1_1_43) to 28 404 (*E.coli*). We simulated reads from 507 genomes from the same species (or strain, if known) as the SPGC reference genomes, and mapped these reads to the SPGC using Bowtie2 (Langmead and Salzberg, 2012). As expected, the rate of assigning reads to a species was correlated with the number of representative genes for the species (Supplementary Fig. S3). A possible confounding factor might be the fraction of reads that map ambiguously to multiple species; however, the median fraction of multi-mapped reads was only 3% across species. Only 129 of the 201 species in the SPGC had an assignment rate of 90% or higher, i.e. 90% of the reads originating from these genomes would be assigned a correct species-level taxonomic label. At one extreme, *Escherichia* sp. 1_1_43, had the lowest number of representative genes and the lowest assignment rate at 2%. Despite having a large number of representative genes, *E.coli* only had an assignment rate of ∼83%, because of the large number of closely related species in the SPGC. All four *Shigella sp.* within the SPGC had low assignment rates: 17%, 11%, 8% and 7% for *S.flexneri*, *S.dysenteriae*, *S.boydii*, *S.sonnei*, respectively. This is because the reads from *Shigella sp.* often map to clusters with an *E.coli* representative sequence.

Due to the importance of *Shigella* sp. for human health, we further analyzed 20 known virulence/toxin genes of *S.sonnei* (Lamba *et al*., 2016; Mattock and Blocker, 2017; Nyholm et al., 2015) (Supplementary Table S2). Only 11 of the 20 genes were taxonomically labelled as *Shigella*, seven were labelled as *Escherichia* and two, *set1A* and *set1B*, were not found at all. Notably, Shiga toxins *Stx1A* and *Stx1B* are labelled as *Escherichia*, even though they are part of a mobile prophage genome which has been horizontally transferred among many *Enterobacteriaceae* (Juhas, 2015), highlighting the difficulty of annotating a mobilome.

### 2.4 Using the IGC as a reference for metagenomic analyses—simulated data

The primary strategy for using the IGC as a reference when analyzing metagenomic datasets involves mapping sequencing reads to the representative sequences of the clusters. Although a seemingly straightforward bioinformatics task, the selection of mapping tools, parameters of the mapping process and characteristics of the reads themselves (e.g. read length) may have a significant impact on the results. To evaluate the effects of such features on the use of the IGC for metagenomic analysis, we simulated three metagenomic samples composed of the species in the SPGC. Two samples simulated Illumina reads (100 nt, 250 nt), and the other simulated 454/IonTorrent reads (225 nt). We compared mapping statistics for tools that are widely used in metagenomic analyses, BWA-MEM (Li and Durbin, 2009) and Bowtie2 (Langmead and Salzberg, 2012) with default parameters, and BLASTN (Altschul *et al*., 1990) with thresholds of 95% identity, 90% read coverage and default values for all other parameters (Table 1).

**Table 1.** The percent of simulated Illumina and 454 Roche reads, from 507 prokaryotic reference genomes, that map to the IGC with BWA-MEM, Bowtie2 and BLASTN

Read Datasets	BLASTN	Bowtie2	BWA-MEM
Illumina 100 nt	74.31	86.44	96.22
Illumina 250 nt	43.98	76.49	98.97
454 Roche ∼225 nt	64.48	77.82	98.18

*Note*: For BLASTN, only those alignments with ≥95% identity and ≥90% read coverage are considered. BWA-MEM and Bowtie 2 were run with default parameters requiring full length matches. Note: 225 nt is the mean length of the 454 Roche reads.

The fraction of reads mapped by different tools, and across different read lengths, varied substantially (Supplementary Table S3). BLASTN consistently mapped fewer reads than the other tools. The gene abundance profiles estimated from these mappings differed significantly across different mapping tools (Mann Whitney U test, *P*-value < 0.001) at every read length, suggesting the choice of mapping tool may confound abundance estimates and, therefore, the associations derived from the data (Supplementary Table S4). Furthermore, nearly half of the reads multi-mapped, i.e. mapped equally well to multiple IGC clusters. Multi-mapped reads can confound taxonomic classification and estimates of abundance, as previously highlighted in RNA-seq studies (Li *et al*., 2010). Our results suggest the need for abundance estimation algorithms that can account for mapping ambiguity (Bray *et al.*, 2016; Patro *et al.*, 2014, 2017), which are rarely used in metagenomic studies.

Together, multi-mapped reads and the poor visibility of some species within the catalog, led to ∼20% of the reads mapping to gene clusters classified as a different genus than that from which the reads originated (Supplementary Table S5). This raises concerns about the accuracy of taxonomic profiles derived from real metagenomic data given that these reads were generated from the genomes used in the construction of the IGC.

### 2.5 Using the IGC as a reference for metagenomic analyses—real data

In addition to the read mapping artifacts discussed previously, genes that are not represented in the IGC but are present in a sample can confound the analysis of metagenomic data. Prior studies have demonstrated the IGC is not a comprehensive representation of the diversity of the human gut microbiome, lacking many genes found in the gut of infants (Bäckhed *et al*., 2015), patients suffering from various diseases such as gout (Guo *et al*., 2016) or diabetes (Forslund *et al.*, 2015), adults from India (only 61% of their gene catalog mapped to the IGC) (Dhakan *et al*., 2019), and even adult twins from the UK (in which a putative 1.5 million genes were not present in the IGC) (Xie *et al*., 2016).

To investigate how read mapping artifacts and genes not represented in the catalog impact analyses based on the IGC, we used a human gut sample from a 61-year-old Cameroonian male with a hunter gatherer diet (SRA accession ERR2619707) (Lokmer *et al*., 2019). We assembled the data with MEGAHIT (Li *et al.*, 2015) and predicted genes using Prokka (Seemann, 2014). Only 66.6% of the predicted genes from this sample clustered to an IGC gene representative, genes to which we refer as the *clustered predicted genes*. The other genes predicted from the sample could not be confidently assigned to IGC clusters (and thus are likely not represented in the IGC), and we refer to these genes as the *unclustered predicted genes*.

**Fig. 4.**
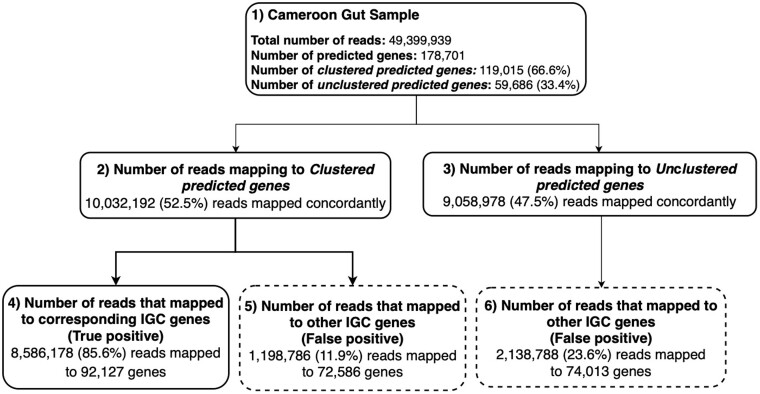
Genes from taxa in samples not represented in the IGC generate noise during analyses with the IGC. Analysis of reads from Cameroonian human gut metagenome sample. Box 1 shows the general statistics of the sample. 66.6% of the predicted genes could be assigned to IGC gene clusters—clustered predicted genes. 33.4% of the predicted genes could not be confidently mapped to the IGC clusters—unclustered predicted genes. 19 091 170 reads mapped concordantly to the predicted genes (52.5% to the clustered predicted genes and 47.5% to the unclustered predicted genes). Among the 10 032 192 reads that mapped concordantly to the clustered predicted genes (Box 2), 11.9% mapped to a different IGC gene than expected (false positives denoted by dashed line Box 5). Of the 9 058 978 reads that mapped concordantly to the unclustered predicted genes (Box 3), 23.6% mapped to IGC genes (false positives denoted by dashed line Box 6)

We separately mapped the reads from the Cameroon dataset with Bowtie2 to the two sets of genes predicted from the sample and the IGC clusters, respectively (Fig. 4). The percent of reads mapping to the predicted genes and the IGC was similar (59.0% to the predicted genes and 55.3% to the IGC), but the percent of multi-mapped reads was much higher for the IGC (24.1%) compared to the predicted genes (3.8%). The reads also mapped to an order of magnitude more IGC clusters (1 369 981) than predicted genes (177 745). Together this suggests a high false positive rate, i.e. that reads from unclustered predicted genes are mapping to IGC clusters representing potentially unrelated genomic sequences and/or functions.

To determine the IGC clusters to which the reads from the clustered predicted genes and the unclustered predicted genes were aligned, we focused our analysis on the read pairs that mapped concordantly to both the predicted genes and to the IGC clusters (24.1% of all reads). A read pair is considered concordantly mapped when the forward and reverse reads of the pair map to a gene with the correct insert size and orientation. Such concordant mappings are less likely to represent mapping artifacts. Given that each clustered predicted gene has a corresponding IGC cluster, we would expect the reads mappings to also be shared between the gene and the cluster to which it is related. Among the 10 032 192 reads that concordantly mapped to clustered predicted genes, 11.9% mapped to a different IGC gene than expected. Conversely, we would expect few read pairs which map to the unclustered predicted genes to map to any IGC clusters given that these genes do not share sufficient similarity with any IGC cluster. Of the 9 058 978 reads that concordantly mapped to the unclustered predicted genes, 23.6% mapped to IGC genes (Fig. 4).

### 2.6 Analysis of other gene catalogs

A survey of 24 gene catalog studies from the last few years highlights that many were created using a similar clustering algorithm as the IGC and thus likely share many of the same issues as those identified above (Table 2). While none of these catalogs provided all the necessary metadata and intermediate files to perform the same analyses as done for the IGC, we were able to predict which issues likely affect the catalogs based upon the description of the methods used to construct these catalogs. We note that 5 of the 24 catalogs were affected by transitive clustering error. Additionally, at least 15 catalogs allowed genes of highly divergent lengths to be clustered together. Further, taxonomic inconsistency and hidden species also likely affect 23 of the catalogs.

**Table 2.** A non-exhaustive list of microbial gene catalogs created in the last few years, and the issues—identified in our analysis of the IGC—that likely affect them based upon a review of the written methods

Gene catalog	Year published	Transitive clustering error	Clusters sequences of highly different lengths	Taxonomic inconsistency	Hidden species	Clustering criteria
Human gut (cirrhosis) (Qin *et al*., 2014)	2014	Yes	Yes	Yes	Yes	– Pairwise comparison of all genes with BLAT: >95% identity and >90% of the shorter gene length. – Merged genes from three catalogs using the same clustering technique.
Mouse gut (Xiao *et al.*, 2015)	2015	No	Unclear	Yes	Yes	Pairwise comparison of all genes with BLAT: >95% identity and overlap >90%
Human gut (infants) (Bäckhed *et al.*, 2015)	2015	No	Yes	Yes	Yes	Pairwise comparison of all genes with BLAT: >95% identity and >90% of the shorter gene length
Human gut (diabetes) ( Forslund *et al*., 2015 )	2015	No	Yes	Yes	Yes	Predicted protein-coding genes with a minimum length of 100 bp were clustered at 95% sequence identity using CD-HIT with parameters set to: -c 0.95, –G 0 -aS 0.9, -g 1, -r 1.
Pig gut (Xiao *et al.*, 2016a)	2016	No	Unclear	Yes	Yes	Pairwise comparison of all genes with BLAT: >95% identity and overlap >90%
Human gut (gout) (Guo *et al.*, 2016)	2016	No	Yes	Yes	Yes	Genes were clustered with CD-HIT using a sequence identity cut-off of 0.95 and a minimum coverage cut-off of 0.9 for the shorter sequences.
Human gut (diabetes) (Xie *et al.*, 2016)	2016	Yes	Yes	Yes	Yes	– Genes were clustered using CD-HIT of the MOCAT pipeline (95% identity, 90% overlap) – Merged the gene set with the IGC catalog using CD-HIT
Chicken gut (Huang *et al.*, 2018)	2018	Unclear	Yes	Yes	Yes	Gene catalog was constructed using CD-HIT-EST with parameters set to: -c 0.95 -n 10 -G 0 -aS 0.9
Rat gut (Pan *et al.*, 2018)	2018	No	Yes	Yes	Yes	Gene ORFs were clustered using CD-HIT with a criterion of 95% identity >90% of the shorter ORF length with default parameter except ‘-G 0 –n 8 –aS 0.9 –c 0.95 –d 0 –g 1’
Dog gut (Coelho *et al.*, 2018)	2018	No	Unclear	Yes	Yes	Genes were clustered at 95% identity using CD-HIT
Macaque gut (Li *et al.*, 2018)	2018	No	Unclear	Yes	Yes	Pairwise comparison of all genes using CD-HIT with identity of >95% and overlap of >90%
Human gut (children) (Vatanen *et al*., 2019)	2018	Yes	Yes	Yes	Yes	– Clustered gene based on sequence similarity at 95% identity and 90% coverage of the shorter sequence using CD-HIT – Merged with the IGC using the same CD-HIT clustering technique to form a comprehensive catalog
South China soil (Lou *et al.*, 2019)	2019	No	Unclear	Yes	Yes	Nucleic acids longer than 100 bp were translated into amino acid sequences. Pairwise comparison of all genes using CD-HIT with parameters >95% identity and >90% overlap
Pig gut (Wang *et al*., 2019)	2019	Yes	Unclear	Yes	Yes	– Predicted genes were clustered at the nucleotide level using CD-HIT with >95% identity and >90% overlap – Combined the catalog with an earlier Pig gut catalog to create a comprehensive catalog
Human lung (Dai *et al.*, 2019)	2019	No	Yes	Yes	Yes	Genes with a length ≥100 bp and without Ns (unidentified nucleotides) were selected to construct non-redundant gene sets using CD-HIT with criteria of >95% identity and >90% alignment of shorter sequence (–c 0.95 –aS 0.9)
Human gut (Indian cohort) (Dhakan *et al.*, 2019)	2019	Yes	Unclear	Yes	Yes	– Pair-wise alignment of genes using BLAT and the genes that had an identity >95% and alignment coverage >90% were clustered into a single set of non-redundant gene. – The gene catalog constructed from Indian samples was combined with the IGC to construct a non-redundant gene catalog (using identity ≥95% and alignment coverage ≥90%)
Rat gut (Zheng *et al*., 2019)	2019	No	Yes	Yes	Yes	Predicted ORFs were clustered using CD-HIT with criteria of >95% identity and >90% alignment of shorter ORF. (-c 0.95, -G 0, -aS 0.9, -g 1, -d 0)
Panthera gut (Mittal *et al.*, 2020)	2020	No	Yes	Yes	Yes	Predicted genes from contigs and from the top abundant microbial species were clustered using CD-HIT using a sequence identity cutoff of 0.95 and minimum coverage cutoff of 0.9 for shorter sequences
Cow gut (Li *et al.*, 2020)	2020	No	Yes	Yes	Yes	Predicted genes were clustered using CD-HIT with ≥95% identity and ≥90% overlap of the shorter sequence (-n 8 -d 0 -g 1 -T 6 -G 0 -aS 0.9 -c 0.95)
Mouse gut (Lesker *et al.*, 2020)	2020	No	No	Unclear	No	Predicted ORFs were taxonomically annotated at different levels, i.e. ORF, contig and bin
Human dental caries (Liu *et al.*, 2020)	2020	No	No	Yes	Yes	Predicted ORFs were clustered using CD-HIT default parameters
Human vagina (Ma *et al.*, 2020)	2020	No	Yes	Yes	Yes	Genes and gene fragments that were at least 99 bp long, with greater than 95% identity over 90% of the shorter gene length were clustered together by CD-HIT-EST
Rhizosphere soil (Zhou *et al*., 2020)	2020	No	Yes	Yes	Yes	Predicted genes were clustered using CD-HIT with >95% sequence identity
Sheep rumen (Ghanbari Maman *et al.*, 2020)	2020	No	Yes	Yes	Yes	CD-HIT tool with the similarity threshold of 95% was used to remove redundant genes

*Note*: The columns of the table list: (i) the gene catalog; (ii) the year it was published; (iii) if the clusters are affected by transitive clustering error; (iv) if the clusters contain sequences of highly different lengths; (v) if the clusters contain sequences from different species; (vi) if species have genes hidden by the genes of other species; (vii) the clustering criteria employed to create the catalog.

## 3 Discussion

Gene catalogs help organize the vast volumes of data generated in metagenomic experiments. If carefully constructed, they provide a valuable resource for the analysis of metagenomic samples. Through our analysis of the IGC—one of the largest gene catalogs available to scientists today—we have highlighted how the design and construction of a gene catalog can affect downstream analyses in unintended ways. These issues affected a large percent of the gene catalogs we found in the literature because many were constructed using similar methods as the IGC.

Perhaps the most prevalent and important source of error for gene catalogs is caused by clustering gene sequences with a fixed threshold, creating clusters composed of sequences with variable levels of taxonomic relatedness. Our observation recapitulates the finding that no specific sequence similarity threshold can be used to consistently capture a particular taxonomic level or functional category. This finding has been well documented previously in the context of 16S rRNA sequencing (Nguyen *et al.*, 2016; Shah *et al.*, 2018). Clustering in this manner effectively hides the taxonomic origin of all but the gene sequences selected as cluster representatives. As a result, each species in a catalog might have a different proportion of genes that are not represented (that are hidden by the genes of other species), genes that are represented once and genes that are represented in multiple copies. This can introduce bias in downstream analyses that aim to explore the presence or abundance of taxa across samples, a bias already noted in the community (McLaren *et al*., 2019). For example, if a catalog contains multiple variants of a gene from a species, metagenomic reads from that gene and species might map to multiple variants in the catalog either uniquely or by multimapping. Through our analysis of the hidden species of the SPGC and the *E.coli* core genes, we have shown that this effect is non-uniform across taxonomic groups and can result in the biased recruitment of reads across taxa.

Another common source of error for gene catalog construction is the clustering of genes of widely different lengths. This can result in clusters where there is little or no overlap between cluster members. While it is not currently possible to confirm the functional consistency of all clusters in a gene catalog, if cluster members share little sequence similarity with the representative (which is treated as the functional homolog of all cluster members) it is likely that they do not share the same function. Furthermore, assessing the relationship between sequence and functional similarity is non-trivial (Ellens *et al.*, 2017) even in the absence of the confounding information introduced by the co-clustering of sequences with widely divergent lengths.

The iterative clustering of catalogs can further exacerbate all of the previously mentioned issues by amplifying the differences between sequences assigned to a cluster. Among the gene catalogs we have explored (Table 2), the use of a multi-step clustering process is typically used for two purposes: to mitigate computational costs, and/or to update an old catalog by merging it with a newer one. However, none of the studies we analyzed took into account the amount of error introduced by iterative clustering. It is certainly desirable to develop computationally efficient catalog construction methods as datasets increase in size, as well as to efficiently incorporate new data into existing catalogs. Our analysis, however, suggests that it is important to ensure that the fidelity of the clusters is not impacted by computational convenience, and highlights the need for additional research in this field.

Coupled with the issues arising from the structure of the clusters themselves, we have shown that the use of the IGC to analyze a real metagenomic sample induces many analytical artifacts, including a high false positive rate—IGC clusters that are not actually found in a sample, but which ‘recruit’ many reads nonetheless. Conversely, as the number of species and the number of their gene variants represented in the catalog increases, so will the number of reads that map ambiguously (Nasko *et al*., 2018). As a result, using gene catalogs that are constructed similarly to the IGC for metagenomic studies will likely introduce analytical artifacts that outweigh the benefit of the common frame of reference these catalogs provide.

While raising these concerns, we agree with the authors of the IGC that properly constructed gene catalogs can be an effective reference for metagenomic studies. However, to maximize their usefulness, gene catalogs should either be created directly from the samples being analyzed or from closely related samples. Our findings indicate that the goal of tracking individual clusters across studies is not met by the IGC and other similarly constructed catalogs. We believe that universal taxonomic identifiers and gene ontologies represent a better approach for relating findings across gene catalogs and metagenomic studies. For gene catalogs to be used as global resources for metagenomic data analysis, new methods for updating catalogs and accounting for biases introduced by read mapping tools needs to be researched. For now, we believe the best use case for gene catalogs is within the narrow context of the samples used to create them.

Our results highlight pitfalls that need to be avoided when constructing such catalogs and reveal several best practices:

- The iterative integration of clusters should be avoided as it amplifies the errors inherent to the clustering process. A multi-step clustering process may be necessary to mitigate computational costs, however we recommend limiting the number of rounds and accounting for the growth in cluster diameter that is due to the multi-round process.
- Arbitrary similarity thresholds should be avoided, and instead researchers should use approaches that are able to dynamically tune clustering parameters (Callahan *et al.*, 2016; Hao *et al.*, 2011; Navlakha et al., 2010; Shah *et al.*, 2018; White *et al*., 2010).
- The clustering procedure should ensure all sequences within a cluster are of similar length.
- The construction of gene catalogs should not exclusively rely on data from metagenomic experiments, but rather should be augmented with genomic sequences from organisms that are commonly found at low abundance in the samples of interest (including eukaryotes and viruses), as such organisms are unlikely to be assembled sufficiently well within the metagenomic data.
- The alignment of sequences to the catalog, as well as estimation of gene abundances from the alignments, should be conducted in a way that adequately addresses non-specific mapping. Several approaches have been developed for RNA-seq analysis that effectively handle multi-mappings in an alignment-free manner (Bray *et al.*, 2016; Patro *et al.*, 2014, 2017), though it remains to be seen whether these are sufficiently effective in metagenomic settings or whether the underlying algorithms need to be adapted.

During the preparation of our manuscript, a new catalog was published (Almeida *et al.*, 2021), which partly addresses some of the issues we have highlighted above. The underlying data being clustered were derived from cultured genome sequences and metagenome-assembled sequences, potentially ensuring a higher quality protein catalog (the Unified Human Gastrointestinal Protein catalog). Gene-level clustering was performed at the protein level in one round of clustering, thereby avoiding transitive clustering error. Notably, the authors of this new study re-clustered the genes from the IGC and appear to be unaware of the blow-up in divergence caused by the iterative process used by the IGC: ‘We clustered the IGC only at 90% and 50% protein identity, as it was originally de-replicated at 95% nucleotide identity’ (Almeida *et al.*, 2021). The Unified Human Gastrointestinal Protein catalog was provided as multiple catalogs constructed with different similarity thresholds, acknowledging that no threshold is appropriate for all analyses. Some of the pitfalls identified above, however, still apply to the new catalog. When clustering protein sequences, Almeida *et al.* only control the fraction of the clustered sequence that needs to match the cluster representative (80% in this case), raising the possibility of artifacts such as that highlighted in Supplementary Figure S2. Furthermore, the new catalog includes the Unified Human Gastrointestinal Genome catalog which is constructed in a two-step process to address the computational cost of clustering. The paper does not indicate that the authors are aware of the additional sequence divergence introduced by this process.

A full-fledged analysis of the new catalog, similar to what we have described above, is beyond the scope of this manuscript. However, as discussed here, it is apparent that issues such as those we have described are not widely appreciated in our community. We hope that our manuscript provides readers with an appreciation for the complexity of sequence clustering, particularly as it relates to metagenomic sequence analysis, and leads to a more thoughtful consideration of the pitfalls we have identified when using gene catalogs as a reference for data analysis.

## Supplementary Material

btab216_supplementary_data
