# Supplementary material for: A critical assessment of gene catalogs for metagenomic analysis
Source: Bioinformatics. 2021 Apr 1;37(18):2848–57. doi: 10.1093/bioinformatics/btab216 (PMC8479683; doi:10.1093/bioinformatics/btab216)
Supplement: btab216_supplementary_data [file btab216_supplementary_data.zip › Supplementary-file-1.pdf]

Transitive clustering error

Clustering a large number of sequences can require impractical amounts of computing time and memory. One technique for addressing the computational cost of clustering uses a divide and conquer paradigm: disjoint subsets of the sequences are clustered separately, then the cluster representatives are clustered together in one or more additional rounds of clustering. When representative sequences from different subsets are clustered together, all members of the corresponding clusters implicitly become part of the resulting cluster. In the IGC, this process was conducted in three rounds. Sequences from each of three distinct geographic regions (American, Chinese, and European) were clustered separately, as were sequences extracted from isolate genomes within the NCBI and EMBL databases, resulting in four distinct catalogs: AGC, CGC, EGC, and SPGC, respectively. In a second round, the three geographically defined catalogs were clustered together, yielding a new catalog, 3CGC, which were then clustered together with the SPGC in a third round of clustering. Each round of clustering used the same cut-offs for the percent identity between a sequence and the cluster representatives, and for the fraction of the sequence that needs to align to the cluster representative in order for it to be assigned to a cluster.

In the following, we discuss the implications of using such an iterative clustering process on the size of the resulting clusters. We focus on two measures of the "tightness" of clusters: the radius (maximum distance between a sequence and the cluster representative); and the diameter (maximum distance between two sequences within a cluster). For the purpose of this discussion, we ignore the impact of partial alignments between a sequence and the cluster representative, and focus exclusively on percent identity as a measure of distance between sequences.

The percent identity cut-off provided to CD-HIT controls the radius of the clusters. After a single round of clustering, the maximum effective radius, R, of the clusters is exactly the same as the cut-off, r, that was given as a parameter to CD-HIT. The maximum effective diameter, D, is exactly 2r. Below, we will show that, with each round of clustering, both the maximum effective radius and diameter of the resulting clusters increases despite using the same cut-off, r, when clustering the representative sequences of clusters generated in a prior round. We call this unintended increase in the effective radius and diameter of clusters *transitive clustering error*.

A general formulation for transitive clustering error

We will start by assuming a set of clusters already constructed, C_1, C_2, …, C_n, which have the effective radii R_1, R_2, …, R_n. We explore here the impact of clustering together the representative sequences of the clusters contained in C_1, C_2, …, C_n, as defined by the effective radius and diameter of the resulting clusters. Before we proceed, it is important to note that our analysis focuses on the worst-case scenario, i.e., we show that it is possible that at least one of the resulting clusterings can have the values for R and D as defined

below. Whether such a worst-case situation may occur depends on the characteristics of the data.

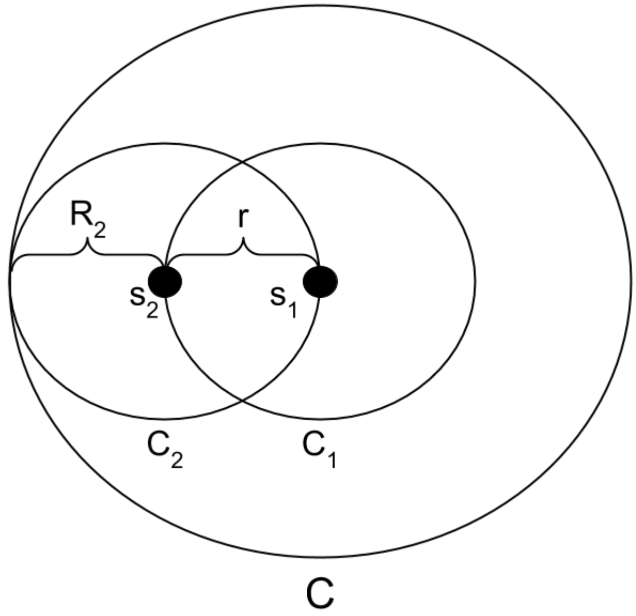

Figure 1: An example cluster, C, with the maximum possible effective radius when clustering the representatives from C_1, C_2, …, C_n with tolerance r.

Lemma 1: The maximum effective radius, R, of the resulting clusters is,

$R = \max(R_1, R_2, \ldots, R_n) + r$

Proof: Refer to figure 1. Without loss of generality, we can assume one resulting cluster, C. By definition, the representative sequence of this cluster must be the representative sequence of one of the clusters in C_1, C_2, …, C_n. Without loss of generality we assume that this is the same as the representative sequence s_1 of the cluster C_1. Further, assume that there is another cluster whose representative sequence was clustered together with s_1. This cluster cannot come from the same catalog as C_1 since its representative sequence is within distance r of s_1 and thus would have been clustered with s_1 already, and therefore could not have seeded its own cluster. Without loss of generality, we can assume that the second cluster is C_2 and that its representative sequence is s_2. To define the radius R of the cluster C we need to compute the maximum distance between a sequence within the selected cluster and its representative s_1. Without loss of generality, let us assume that $R_2 = \max(R_1, ..., R_n)$. Given the above, the maximum distance between a sequence within the cluster defined by s_1 and s_1 is the sum of r, the maximum distance between s_1 and s_2, and R_2, the maximum distance between a sequence within the cluster defined by s_2 and its cluster representative, thereby proving the lemma.

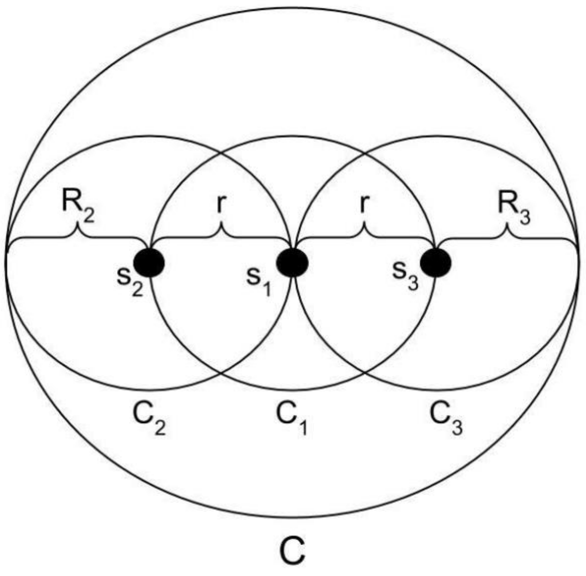

Figure 2: An example cluster, C, with the maximum possible effective diameter when clustering the representatives from C_1, C_2, …, C_n with tolerance r.

Lemma 2: The maximum effective diameter D, is,

$D = 2*\max(R_1, R_2, \ldots, R_n) + 2r = 2R$

Proof: Refer to figure 2. The proof follows the same template as that for the radius, except that the selected cluster, C, is assumed to be clustered with an additional cluster, C_3, where $R_3 = R_2 = \max(R_1, ..., R_n)$ and the representative of C_3, s_3, is 2r divergent from s_2.

Given the two lemmas, we can now explore the impact on R and D of the number of iterative clustering steps.

Transitive clustering error in the IGC

The diagram in figure 3 highlights the clustering strategy used by the IGC. At each stage, the clustering cut-off, r, was set to 5% divergence (95% identity). Using the formulas derived above, we demonstrate the increase in effective radius and diameter that occurs at each clustering stage, reaching a maximum radius of 15% for the IGC. In other words, within the IGC it is theoretically possible that a sequence may share as little as 85% identity with the corresponding cluster representative, and two sequences that are co-clustered may share as little as 70% identity with each other. These values exceed the nucleotide identity assumed to define a species for the IGC i.e. 95% identity.

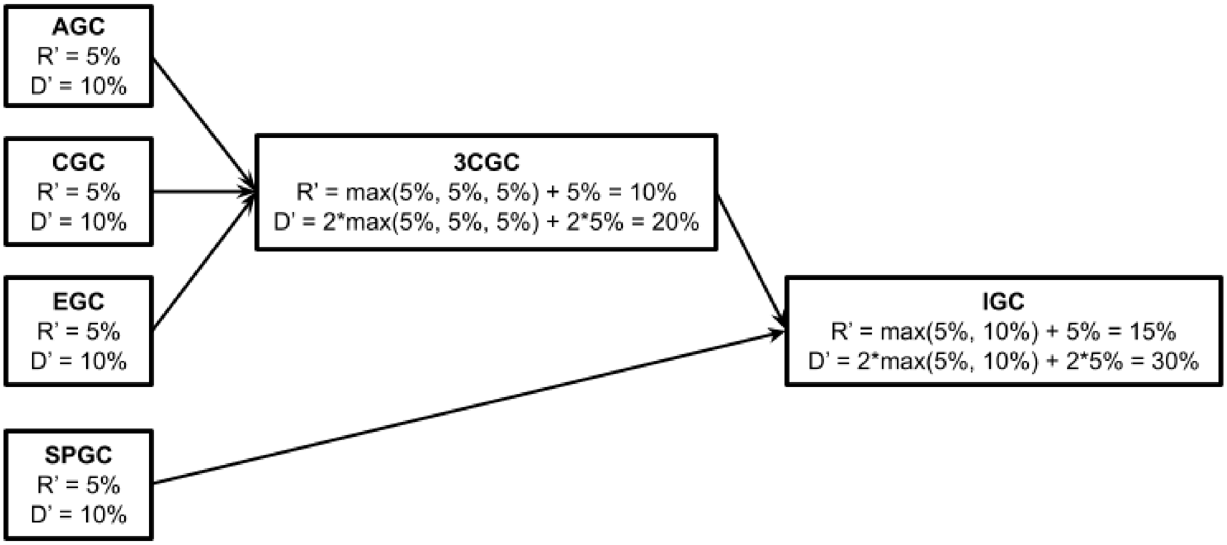

Figure 3: Clustering strategy used in creating the IGC. Each block represents catalogs created in the process, and shows the worst-case radius and diameter of clusters in the catalog.

Impact of clustering strategy on cluster radius

In figure 4 we compare five different approaches for constructing a catalog from four different catalogs. These range from a single round of clustering that joins all catalogs together in one round (A), to a four-round process that iteratively adds an additional catalog to the previously clustered ones (E). The process used by the IGC is in panel (B). As can be seen in the figure, the final effective radius ranges from 2r (A) to 4r (E), demonstrating how different clustering strategies impact the effective radius of clusters in the final catalog.

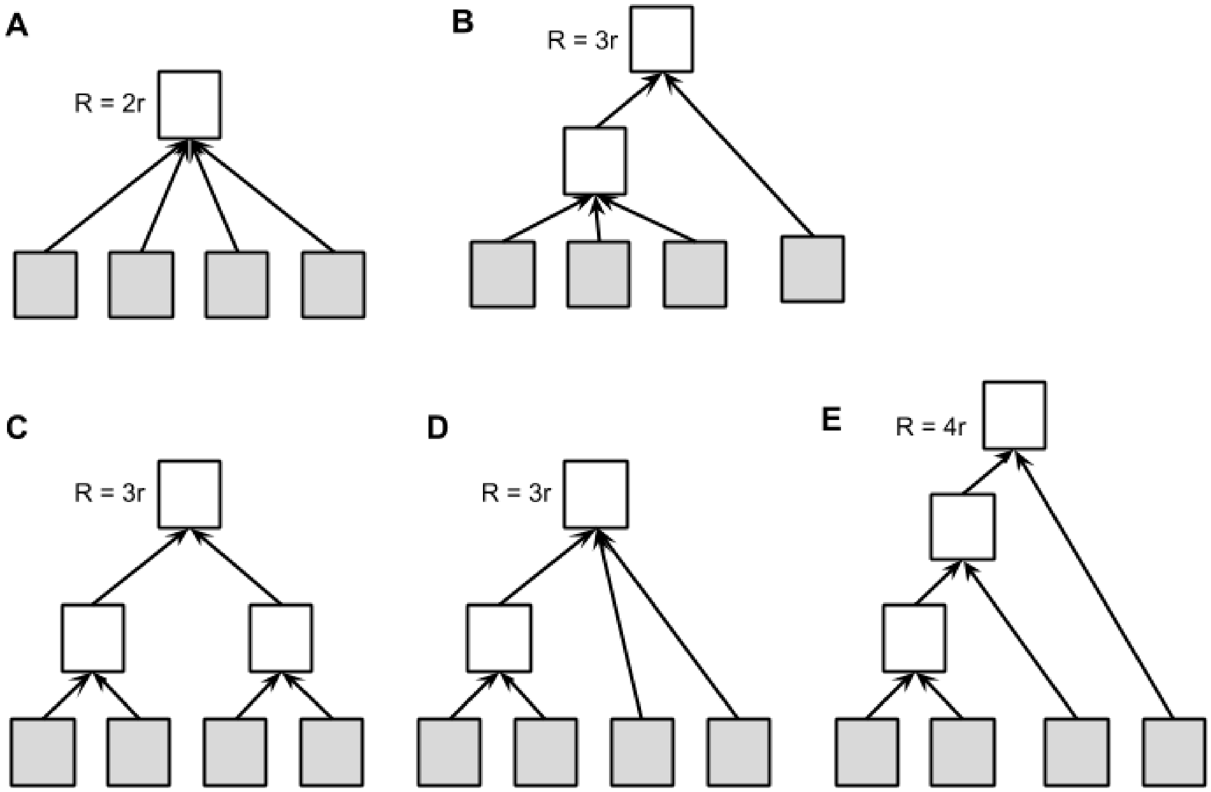

Figure 4: Example topologies to combine sequences from four catalogs (grey squares) into one final catalog. The order of combining catalogs can impact the effective radius and diameter of the clusters in the final catalog. For each topology, the effective radius of the final catalog is displayed.
