# Supplementary material for: A critical assessment of gene catalogs for metagenomic analysis
Source: Bioinformatics. 2021 Apr 1;37(18):2848–57. doi: 10.1093/bioinformatics/btab216 (PMC8479683; doi:10.1093/bioinformatics/btab216)
Supplement: btab216_supplementary_data [file btab216_supplementary_data.zip › Supplementary-file-3.pdf]

Supplementary information

Supplementary Table 1: Domain structure of the representative sequence, MH0244_GL0138579, of Cluster 303 in the IGC as determined by BLASTP alignment with nr database (version 5) on NCBI.

Domain name	Accession	Description	Interval
LPD14	pfam18827	Large polyvalent protein-associated domain 14	476-618
DEXHc_Snf	cd17919	DEXH/Q-box helicase domain of DEAD-like helicase Snf family proteins	587-3682
tolA	PRK09510	cell envelope integrity inner membrane protein TolA	1076-1208
ddrB-ParB	pfam18763	ddrB-like ParB superfamily domain	1281-1404
InPase	pfam18823	Inorganic Pyrophosphatase	1834-2003
COG4646	COG4646	Adenine-specific DNA methylase, N12 class	2918-3449
COG4646	COG4646	Adenine-specific DNA methylase, N12 class	3610-3821
SF2_C_SNF	cd18793	C-terminal helicase domain of the SNF family helicases	3824-3971
HELICc	smart00490	helicase superfamily c-terminal domain	3870-3956
Helicase_C	pfam00271	Helicase conserved C-terminal domain	3885-3956
LPD38	pfam18857	Large polyvalent protein associated domain 38	5237-5421

Supplementary Table 2: Taxonomic annotation of twenty virulence/toxin genes of *Shigella sonnei* when aligned to the SPGC catalog.

**Toxin/Virulence factor**	**Genus of most similar gene in IGC**	**Percent identity of the top BLAST hit in IGC**
ShiA	*Shigella*	100.00
ShiB	*Shigella*	94.41
ShiC	*Shigella*	100.00
ShiD	*Shigella*	100.00
ShiE	*Shigella*	99.43
ShiF	*Shigella*	99.75
ShiG	*Escherichia*	84.44
IucA	*Escherichia*	99.83
IucB	*Escherichia*	99.37
IucC	*Escherichia*	96.38
IucD	*Shigella*	99.78
IutA	*Escherichia*	99.45
Pic	*Shigella*	99.64
GtrA	*Shigella*	99.34
GtrB	*Shigella*	98.15
SigA	*Shigella*	97.67
set1A	Not found	NA
set1B	Not found	NA
Stx1A	*Escherichia*	100.00
Stx1B	*Escherichia*	100.00

Supplementary Table 3: Read mapping statistics for different tools (BLASTN, Bowtie2, BWA-MEM) for the reads simulated by ART simulator for 454 Roche technology and Illumina (100 nt, 250 nt) technology. For BLASTN, only those alignments that have ≥ 95% identity and ≥ 90% read coverage are considered.

**Mapping tool**	**Dataset**	**Unmapped reads**	**Reads mapped exactly once**	**Multi-mapped reads**	**Total reads**
BLASTN	454 Roche 225 nt	12046662 (35.52%)	20607264 (60.76%)	1259937 (3.72%)	33913863
Bowtie2	454 Roche 225 nt	7523531 (22.18%)	12727602 (37.53%)	13662730 (40.29%)	33913863
BWA-MEM	454 Roche 225 nt	615080 (1.81%)	17789182 (52.45%)	15509601 (45.73%)	33913863
BLASTN	Illumina 100 nt	24590586 (25.69%)	63782504 (66.64%)	7339930 (7.67%)	95713020
Bowtie2	Illumina 100 nt	12977730 (13.56%)	42142225 (44.03%)	40593065 (42.41%)	95713020
BWA-MEM	Illumina 100 nt	3618165 (3.78%)	49637777 (51.86%)	42457078 (44.36%)	95713020
BLASTN	Illumina 250 nt	21407600 (56.03%)	16112369 (42.17%)	690019 (1.81%)	38209988
Bowtie2	Illumina 250 nt	8984244 (23.51%)	14631373 (38.29%)	14594371 (38.20%)	38209988
BWA-MEM	Illumina 250 nt	392069 (1.03%)	20811950 (54.47%)	17005969 (44.50%)	38209988

Supplementary Table 4: P-values from Mann Whitney U Test comparing the gene abundance profiles generated by different mapping tools when mapping simulated reads, of varying lengths, to the IGC.

**Read Length**	**BWA-MEM vs Bowtie2**	**BWA-MEM vs BLASTN**	**Bowtie2 vs BLASTN**
Illumina 100 nt	2.68×10^{-251}	1.12×10^{-39}	2.45×10^{-25}
Illumina 250 nt	3.27×10^{-07}	0.0	3.84×10^{-123}

Supplementary Table 5: Read mapping statistics for testing the taxonomic classification performance of the IGC on data simulated from genomes with the same taxonomy as the SPGC reference genomes.

**Read Length**	**Percent of reads mapped to IGC**	**Percent of reads mapped to correct genus**
Illumina 100 nt	86.4	81.7
Illumina 250 nt	76.5	82.1

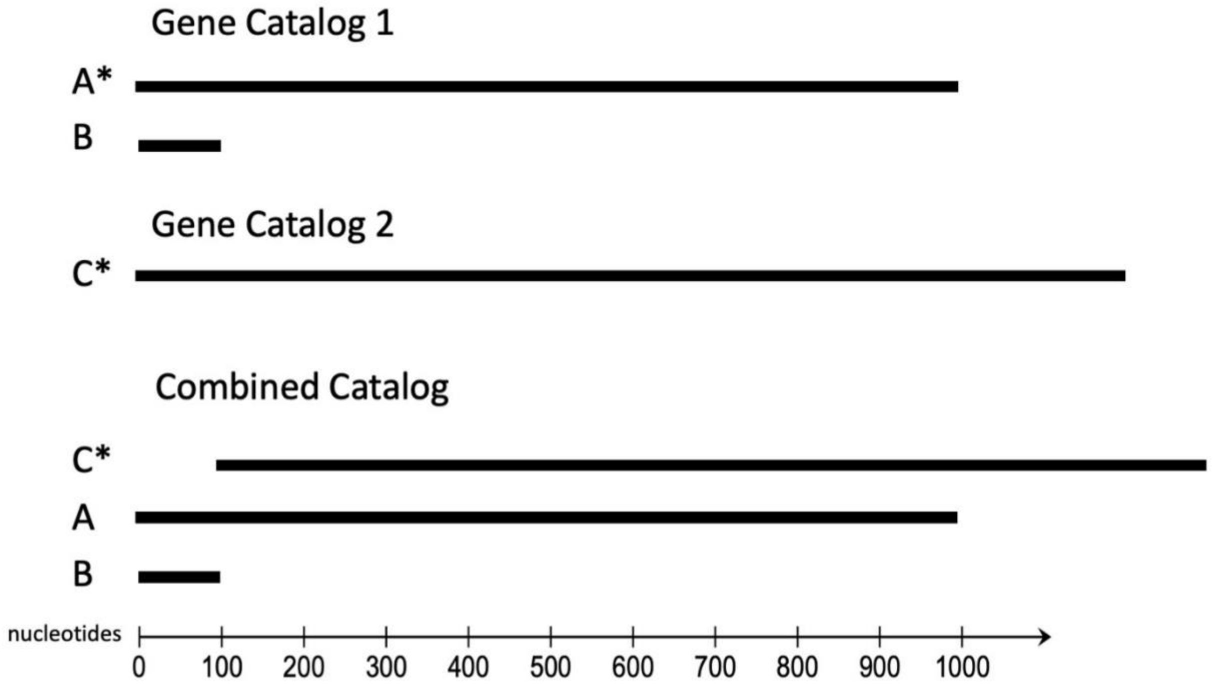

Supplementary Figure 1: A schematic example of how, in a worst-case scenario, clustering separate gene catalogs with CD-HIT can recruit sequences that do not overlap with the representative sequence given the IGC clustering parameters. The sequences within each gene catalog are aligned. Here * denotes the representative sequence of the catalog. Gene A and Gene B were clustered together to create Gene Catalog 1. Gene A is the representative sequence because it is the longest sequence (default of CD-HIT). In this case 100% of the length of Gene B aligns to 10% the length of Gene A with 100% identity. Gene C is a representative sequence in Catalog 2 with no clustered sequences. Gene A and Gene C were clustered to create the Combined Catalog. Gene C becomes the new representative, because it is longer than Gene A, and Gene A and Gene B become cluster members. In the Combined Catalog, 90% of the length of Gene A aligns to Gene C with 100% identity and Gene B has no overlap with Gene C at all.

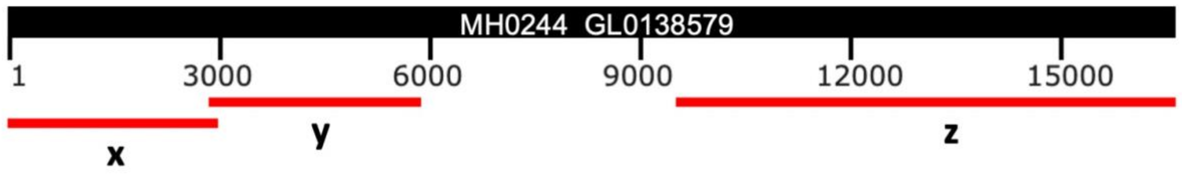

Supplementary Figure 2: BLASTN alignment of the IGC Cluster 303 representative sequence, MH0244_GL0138579 (16,611 nt), and the 3 cluster members x (469585.HMPREF9007_02027, 2,982 nt), y (469585.HMPREF9007_02028, 3,012 nt), and z (469585.HMPREF9007_02029, 7,122 nt). All were predicted as complete genes (from start to stop codon), yet each cluster member only partially aligns to the representative with a small overlap between x and y and no overlap between y and z.

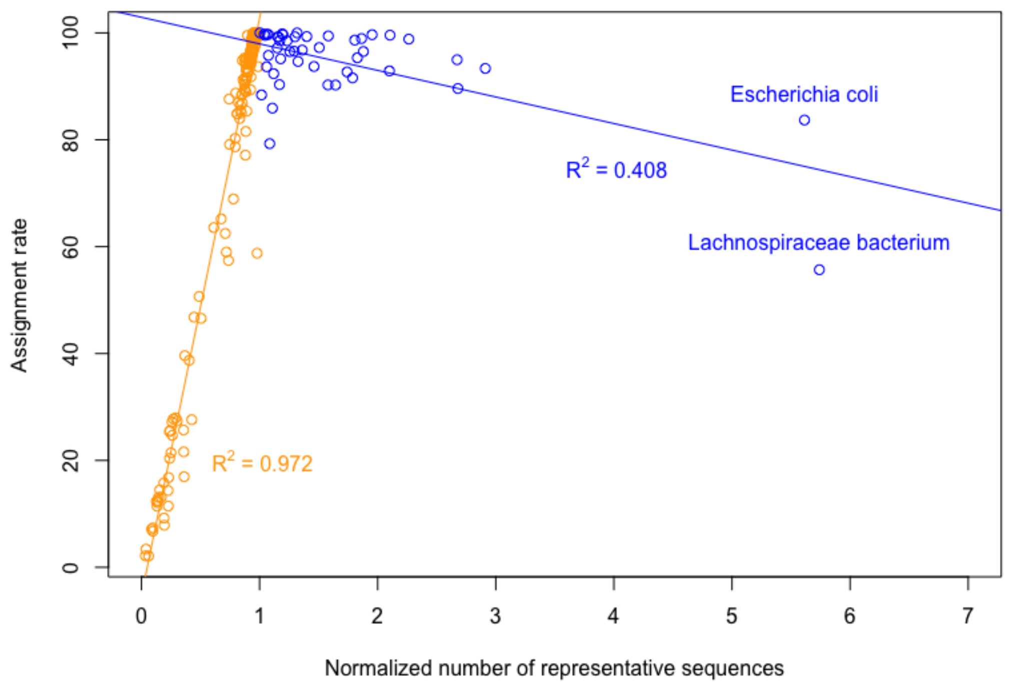

Supplementary Figure 3: The relationship between the number of representative genes (normalized by the mean number of genes per genome) per species and their assignment rate in a simulated metagenomic dataset of the SPGC genomes. The assignment rate is the percent of simulated reads from a species that map to the corresponding representative sequences for that species in the SPGC. For most species in the SPGC, the number of representative genes (normalized by the mean number of genes per genome) is 1 or less (orange). The assignment rate for these species has a positive correlation (orange least squares line) with the number of representatives. For some species, however, the number of representative genes normalized by the mean number of genes per genome can be greater than 1 (blue). These species have genes from multiple genomes and are effectively represented as a pangenome in the SPGC. For example, *E. coli* has 28,404 representative genes and 124 genomes in the SPGC. For these species there is a weak negative correlation (blue least squares line) between the assignment rate and the number of representatives.
