# Supplementary material for: A critical assessment of gene catalogs for metagenomic analysis
Source: Bioinformatics. 2021 Apr 1;37(18):2848–57. doi: 10.1093/bioinformatics/btab216 (PMC8479683; doi:10.1093/bioinformatics/btab216)
Supplement: btab216_supplementary_data [file btab216_supplementary_data.zip › Supplementary-file-4-methods.pdf]

IGC Data

The IGC representative sequences, the corresponding metadata as well as the data (reads, contigs, and predicted genes) used to create it were downloaded from https://db.cngb.org/microbiome/genecatalog/genecatalog_human/ and http://gigadb.org/dataset/100064. From the CD-HIT clustering output files, we were able to associate predicted genes with the corresponding cluster representative for the AGC, CGC, EGC, SPGC, 3CGC, and IGC gene catalogs (provided as IGCcluster_of_cluster.txt via links in Data and Code Availability section).

Transitive clustering error

To detect transitive clustering error in the IGC, 255,191 IGC gene clusters with at least 100 sequences were analyzed. Each cluster was clustered with CD-HIT (version 4.8.1) using two different sets of parameters: 1) The IGC parameters, ≥ 95% identity and ≥ 90% query coverage (-c 0.95 -aS 0.9); and 2) with relaxed parameters, ≥ 50% identity and ≥ 90% query coverage (-n 3 -c 0.50 -aS 0.9). For each cluster, we parsed the CD-HIT output files to identify the cluster member with the minimum percent identity to the representative. If the cluster was split into two or more groups, we note that these clusters had < 50% identity between the representative and the most divergent cluster member.

Clustering Sequences of Divergent Lengths

For 255,191 IGC gene clusters with at least 100 cluster members, we computed the length difference between the shortest and the longest sequence, and the percent of the longest sequence aligned to the shortest sequence.

Taxonomic Homogeneity of Lactobacillus and Bacteroides Gene Clusters

The predicted gene coding sequences from 167 *Bacteroides* species (5,355,696 genes) and 166 *Lactobacillus* species (1,876,284 genes) were downloaded from the NCBI RefSeq database. These sequences were clustered with MMseqs2 (Steinegger et al. 2017) at 95% identity requiring at least 90% of the shorter sequence to align.

Taxonomic Homogeneity of IGC Clusters

We determined the number of species per cluster in the SPGC catalog from the CD-Hit clustering output file provided by the IGC authors. Next, we estimated the number of species in a subset of 200 IGC clusters with at least 100 sequences each (the 100 largest clusters and 100 randomly chosen clusters). All gene sequences belonging to the sampled subset of clusters, were translated from nucleotides to amino acids. We aligned amino acid sequences to the NCBI nr database (version 5) with Diamond (version 0.9.29) BLASTP (≥ 95% identity and ≥ 90% query coverage). Two methods were used to estimate the number of species per cluster, listed here in order from most to least conservative: 1) the minimum set cover of species was identified in the Diamond results such that every sequence within a cluster had one species from the set in its alignment results; 2) the number of unique species was counted per cluster by using the top hit from Diamond result.

Cluster Homogeneity of the Core Genes of *Escherichia coli*

Genome assemblies of *Escherichia coli* (86,830) were downloaded from NCBI via the identifiers provided by the pathogen detection website (https://www.ncbi.nlm.nih.gov/pathogens/isolates/#/search/562). The core genes were extracted from the assemblies to build nonredundant allele databases using the methods described in Pightling et al. (Pightling et al. 2015) and Pettengill et al. (Pettengill et al. 2016). The alleles of each core gene were clustered separately with CD-HIT using ≥ 50% identity and ≥ 90% query coverage (-n 3 -c 0.50 -aS 0.9). We recorded the percent identity between the representative and the most divergent cluster member for each core gene. If CD-HIT split the cluster into two, we noted the percent identity between the representative and the most divergent cluster member as < 50.

Visibility of Species in Gene Catalogs

The number of representative genes per species was counted in the SPGC gene catalog. The taxonomic origin of each gene sequence in the SPGC catalog is known based on the reference genome from which the gene is predicted. To test how the number of representative genes per species contributed to the taxonomic classification of the species in the sample, we used ART (Huang et al. 2012) sequence simulator (version 2.5.8) to simulate 100 nt Illumina reads from 507 genomes downloaded from NCBI that belonged to the same species (or strain, if known) as the SPGC reference genomes (Supplementary File 2). We mapped the reads to the SPGC with Bowtie2 (version 2.3.0, and parameters --no-unal --no-head -

U) and plotted the number of representative genes (normalized by the mean number of genes per genome for that species) per species against the assignment rate for each species. The assignment rate is the percent of simulated reads from a species that map to the corresponding representative sequences for that species in the SPGC. We also quantified the taxonomic classification performance of the IGC by calculating the percentage of simulated reads that mapped to the correct genus, i.e. the same genus as the reference genome from which the read originated. The 20 virulence/toxin genes of *Shigella sonnei* were identified in the IGC by using the best BLASTN hit to the IGC and then retrieving the taxonomy of the best hit from the IGC data.

Using the IGC as a reference for metagenomic analyses — simulated data

We used ART to simulate three datasets from 507 genomes downloaded from NCBI that belonged to the same species (or strain, if known) as the SPGC reference genomes (Supplementary File 2). Two samples simulated single end Illumina reads (100 nt, 250 nt), and the other simulated 454/IonTorrent reads (mean read length 225 nt). We compared mapping statistics for BWA-MEM (version 0.7.5) and Bowtie2 (version 2.3.0) with default settings, as well as BLASTN (version 2.6.0) (with 95% identity and 90% query coverage parameters). The relative abundance for each gene with mapped reads was calculated and the gene abundance profiles estimated from these mappings were compared using a Mann Whitney U test across different mapping tools and read lengths.

Using the IGC as a reference for metagenomic analyses — real data

A single gut metagenome from a 61 year old male Cameroonian hunter gatherer (SRA ERR2619707) was downloaded from NCBI and assembled with MEGAHIT (Li et al. 2015) using default settings. Genes were predicted in the assembly with Prokka (Seemann 2014) (prokka --metagenome). Genes were assigned to the IGC clusters with BLASTN if there was alignment with ≥ 90% query coverage and ≥ 95% identity. The reads were mapped with Bowtie2 (both with and without --no-discordant and --no-mixed), using default settings, to both the IGC and the genes predicted from the assembly. The mean depth of coverage for the IGC genes was calculated as the total number of base pairs mapped to a gene divided by the gene length.

References

Steinegger, Martin, and Johannes Söding. "MMseqs2 enables sensitive protein sequence searching for the analysis of massive data sets." Nature biotechnology 35.11 (2017): 1026-1028.

Pightling, Arthur W., Nicholas Petronella, and Franco Pagotto. "The Listeria monocytogenes Core-Genome Sequence Typer (LmCGST): a bioinformatic pipeline for molecular characterization with next-generation sequence data." BMC microbiology 15.1 (2015): 1-12.

Pettengill, James B., et al. "Real-time pathogen detection in the era of whole-genome sequencing and big data: comparison of k-mer and site-based methods for inferring the genetic distances among tens of thousands of Salmonella samples." PloS one 11.11 (2016): e0166162.

Huang, Weichun, et al. "ART: a next-generation sequencing read simulator." Bioinformatics 28.4 (2012): 593-594.

Li, Dinghua, et al. "MEGAHIT: an ultra-fast single-node solution for large and complex metagenomics assembly via succinct de Bruijn graph." Bioinformatics 31.10 (2015): 1674-1676.

Seemann, Torsten. "Prokka: rapid prokaryotic genome annotation." Bioinformatics 30.14 (2014): 2068-2069.
